# The Antibody Targeting the E314 Peptide of Human Kv1.3 Pore Region Serves as a Novel, Potent and Specific Channel Blocker

**DOI:** 10.1371/journal.pone.0036379

**Published:** 2012-04-27

**Authors:** Xiao-Fang Yang, Yong Yang, Yi-Tian Lian, Zhao-Hui Wang, Xiao-Wei Li, Long-Xian Cheng, Jin-Ping Liu, Yan-Fu Wang, Xiang Gao, Yu-Hua Liao, Min Wang, Qiu-Tang Zeng, Kun Liu

**Affiliations:** 1 Department of Cardiology, Union Hospital, Huazhong University of Science and Technology, Wuhan, China; 2 Department of Geriatrics, Union Hospital, Huazhong University of Science and Technology, Wuhan, China; 3 Department of Cardiology, Tianjin Chest Hospital, Tianjin, China; 4 Department of Cardiovascular Surgery, Union Hospital, Huazhong University of Science and Technology, Wuhan, China; 5 Department of Cardiology, Affiliated Hospital, Jining Medical College, Shandong, China; 6 Department of Geriatrics, Tongji Hospital, Huazhong University of Science and Technology, Wuhan, China; University of Muenster, Germany

## Abstract

Selective blockade of Kv1.3 channels in effector memory T (T_EM_) cells was validated to ameliorate autoimmune or autoimmune-associated diseases. We generated the antibody directed against one peptide of human Kv1.3 (hKv1.3) extracellular loop as a novel and possible Kv1.3 blocker. One peptide of hKv1.3 extracellular loop E3 containing 14 amino acids (E314) was chosen as an antigenic determinant to generate the E314 antibody. The E314 antibody specifically recognized 63.8KD protein stably expressed in hKv1.3-HEK 293 cell lines, whereas it did not recognize or cross-react to human Kv1.1(hKv1.1), Kv1.2(hKv1.2), Kv1.4(hKv1.4), Kv1.5(hKv1.5), KCa3.1(hKCa3.1), HERG, hKCNQ1/hKCNE1, Nav1.5 and Cav1.2 proteins stably expressed in HEK 293 cell lines or in human atrial or ventricular myocytes by Western blotting analysis and immunostaining detection. By the technique of whole-cell patch clamp, the E314 antibody was shown to have a directly inhibitory effect on hKv1.3 currents expressed in HEK 293 or Jurkat T cells and the inhibition showed a concentration-dependence. However, it exerted no significant difference on hKv1.1, hKv1.2, hKv1.4, hKv1.5, hKCa3.1, HERG, hKCNQ1/hKCNE1, L-type Ca^2+^ or voltage-gated Na^+^ currents. The present study demonstrates that the antibody targeting the E314 peptide of hKv1.3 pore region could be a novel, potent and specific hKv1.3 blocker without affecting a variety of closely related K_v_1 channels, KCa3.1 channels and functional cardiac ion channels underlying central nervous systerm (CNS) disorders or drug-acquired arrhythmias, which is required as a safe clinic-promising channel blocker.

## Introduction

Over the last decade, the voltage-gated potassium channel, Kv1.3, with its distribution largely in immunocytes and certain areas in the brain [1], [2], has received much attention and gained a vast body of compelling evidence on its modulation of specified lymphocyte subsets. In autoimmune diseases including multiple sclerosis, type-1 diabetes, psoriasis, rheumatoid arthritis, transplant rejection, graft-versus-host disease, SjÖgren's syndrome, and systemic lupus erythematosus, effector memory T cells contribute greatly to inflamed injuries [3]–[13]. Focusing on the role of Kv1.3 in the modulation of lymphocyte subsets, a series of studies reveal that the presence of Kv1.3 controls activation and proliferation of autoreactive effector lymphocytes [11], [14]–[17]. Inhibition of Kv1.3 channels leads to the down-regulation of TEMs activities, which was validated to ameliorate autoimmune diseases in animal models [18]–[21]. These data suggest that Kv1.3 represents a novel target for the treatment of autoimmune diseases. And as a promising therapeutic approach, selective blockade of Kv1.3 attracts more attention in seeking potent Kv1.3 blockers.

Small molecules or peptide toxins have been explored for selective Kv1.3 blockers [22]–[35], however, quite a few of them lack ion channel selectivity and exhibit a broad pattern of channel blockers [24], [36], [37]. In addition to Kv1.3 blockade, these chemicals block other homologous K^+^ channels as well as Na^+^ or Ca^2+^ channels [27]. Thus blockade of the channels underlies fatal arrhythmias or central nervous systerm(CNS) disorders.

Antibodies have the characteristics of high affinity and specificity. We herein generated the antibody directed against one peptide of human Kv1.3 extracellular loop as a novel and specific Kv1.3 blocker.

## Results

### The E314 antibody generation

**Figure 1:**
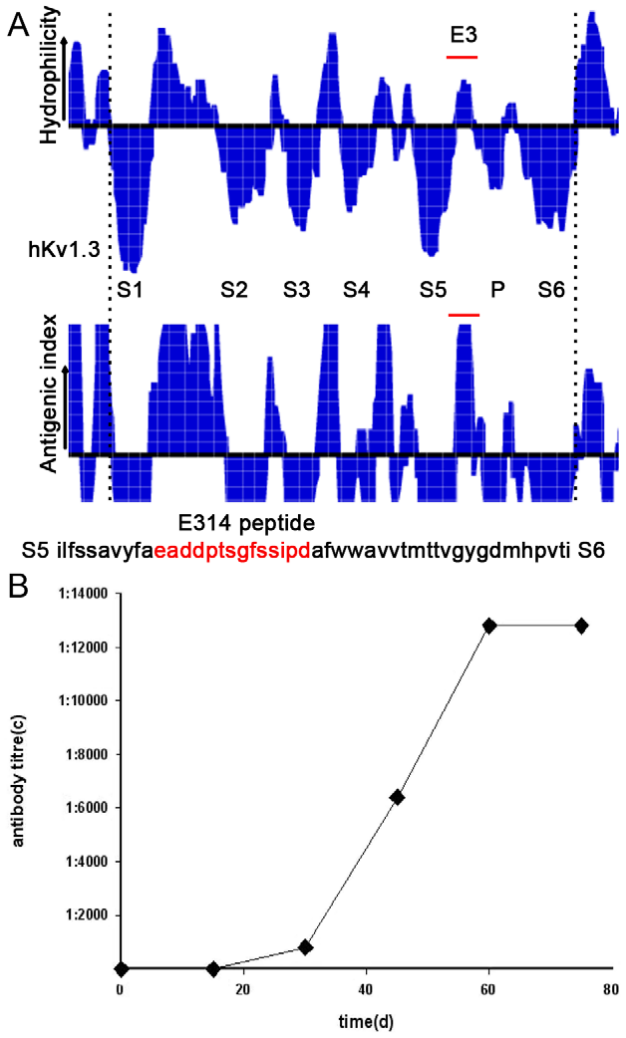
The E314 peptide selection and the E314 antibody generation. (A) Six-membrane spanning (S1–S6) of hKv1.3 channel α subunit and pore region between S5 and S6 was depicted by hydrophilicity analysis of its constituent amino acid aligment. The E314 peptide located at pore region was selected according to amino acid antigenic index. (B) The E314 antibody titre was assayed by enzymelinked immunosorbent assay (ELISA). The E314 antibody wth a high titre was generated after 5 immunizations.

The E314 peptide containing 14 amino acids is located at the external end of hKv1.3 pore region. The amino acid sequence is shown as follows: Glu- Ala- Asp- Asp- Pro- Thr- Ser- Gly- Phe- Ser- Ser- Ile- Pro- Asp (China patent application number:201110044416.X Fig. 1A). By immunizing rabbits with the hapten, we generated the polyclonal antibody against the hKv1.3 E314 peptide with a high titre. After three immunizations, the antibody titre in serum was markedly boosted and reached a high and stable level at the termination of the immunization (Fig. 1B).

### The E314 antibody specifically recognizes or binds to human Kv1.3 protein

**Figure 2:**
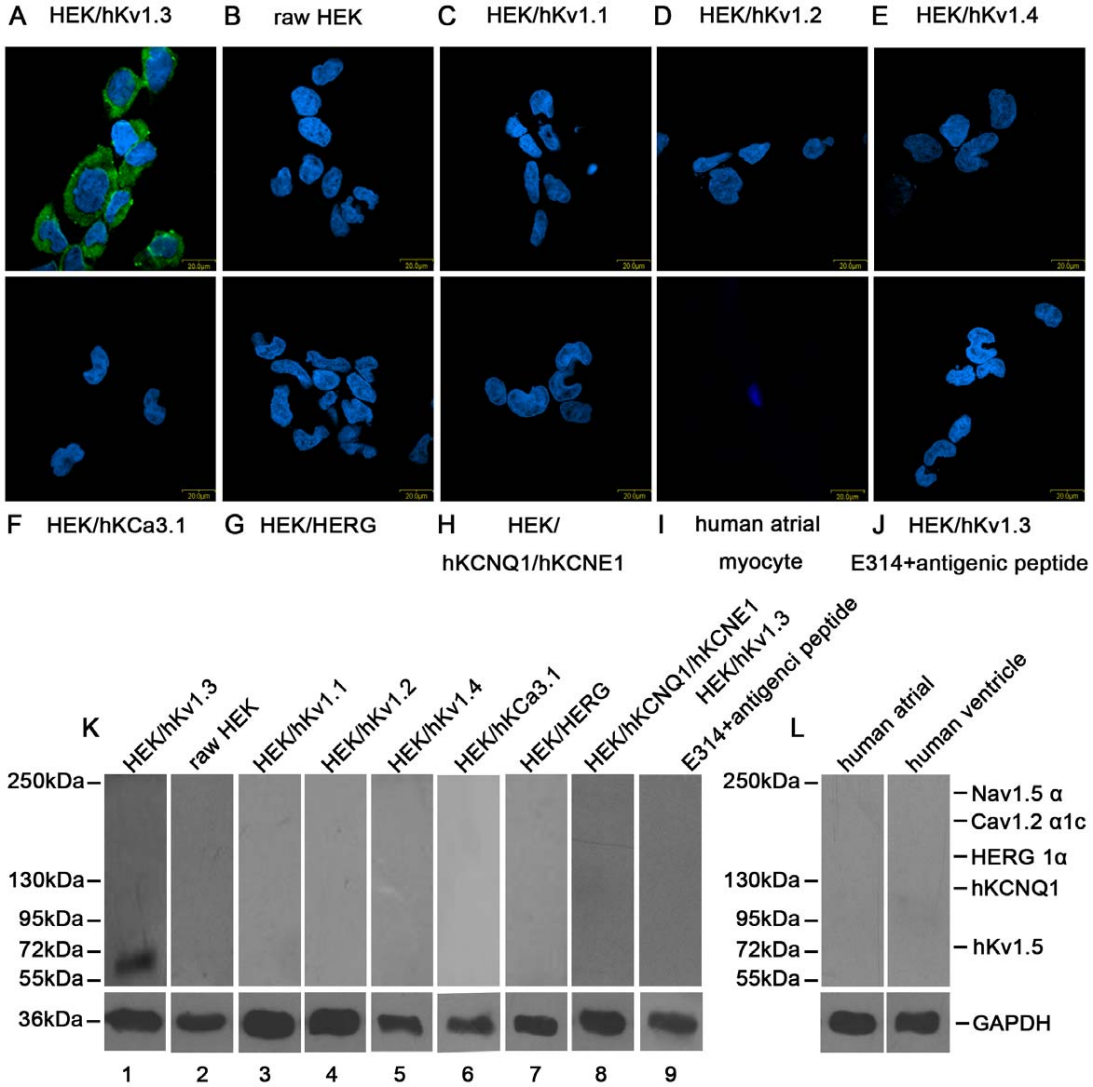
The E314 antibody specific recognition of human Kv1.3 protein by immunostaining and Western blotting. Plasma membrane was stained with green fluorescence in HEK 293 cells stably expressing hKv1.3 channels (A), whereas no membrane fluorescence was detected in raw HEK 293 cells (B), HEK 293 cells stably expressing hKv1.1 channels (C), hKv1.2 channels (D), hKv1.4 channels (E), hKCa3.1 channels (F), HERG channels (G), hKCNQ1/hKCNE1 channels (H), human atrial myocytes (I), or HEK 293 cells stably expressing hKv1.3 channels exposed to the E314 antibody preincubated with an excess of the E314 peptide (J). Nuclei were stained with blue fluorescence by using DAPI labelling. In HEK 293 cells stably expressing hKv1.3 protein, the E314 antibody specifically recognized 63.8KD protein (K lane 1), whereas the recognition was absent in raw HEK 293 cells (K lane 2), HEK 293 cells stably expressing hKv1.1 protein (K lane 3), hKv1.2 protein (K lane 4), hKv1.4 protein (K lane 5), hKCa3.1 protein (K lane 6), HERG protein (K lane 7), hKCNQ1/hKCNE1 protein (K lane 8) or when the E314 antibody was preincubated with an excess of the E314 peptide (K lane 9). In human atrial or ventricular myocytes, the E314 antibody did not recognize 145KD/155KD HERG protein, 120KD hKCNQ1 protein, 220KD Nav1.5 protein or 190KD Cav1.2 protein (L).

By immunostaining, we observed the binding of the E314 antibody diluted at 1∶200 to plasma membranes respectively in raw HEK 293 cells (Fig. 2B), HEK 293 cell lines stably expressing hKv1.3 (Fig. 2A), hKv1.1, hKv1.2, hKv1.4, hKCa3.1, HERG and hKCNQ1/hKCNE1 proteins (Fig. 2C, D, E, F, G and H) and human atrial myocytes (Fig. 2I). The results indicated that there was only green fluorescence detected on plasma membranes in HEK 293 cells stably expressing hKv1.3 channels and the fluorescence signals could be completely blocked by the E314 antibody preincubated with an excess of the E314 peptide (Fig. 2J).

By Western blotting, we analyzed the E314 antibody specific recognition. As illustrated in Figure 2, the E314 antibody showed a specific recognition of hKv1.3 protein. The E314 antibody diluted at 1∶1000 recognized 63.8KD hKv1.3 protein stably expressed in HEK 293 cells (Fig. 2K lane 1), whereas it was not able to recognize identical molecular weight protein in raw HEK 293 cells or 56KD hKv1.1, 70–80KD hKv1.2, 68KD hKv1.4, 45KD hKCa3.1, 145/155KD HERG, 120KD hKCNQ1 protein stably expressed in HEK 293 cells (Fig. 2K lane 2, 3, 4, 5, 6, 7 and 8). Specific recognition of hKv1.3 protein could be completely blocked by the E314 antibody preincubated with an excess of the E314 peptide (Fig. 2K lane 9). Furthermore in human atrial or ventricular myocytes, we demonstrated that the E314 antibody did not recognize 75KD Kv1.5 protein, 145/155KD HERG protein, 120KD hKCNQ1 protein, 220KD Nav1.5 protein or 190KD Cav1.2 protein comprising chiefly of L-type Ca^2+^ channel (Fig. 2L).

### The E314 antibody inhibits human Kv1.3 currents stably expressed in HEK 293 cells

Using the whole-cell patch clamp technique, we tested the ability of the E314 antibody inhibiting hKv1.3 currents stably expressed in HEK 293 cells. The hKv1.3 currents were gradually inhibited after addition of the 300 nM E314 antibody to the external solution and the inhibition reached about 55% steady-state level in 10–15 minutes whcih was not reversible by washout (e.g., Figure S1 for the first supporting information figure). To obtain potent binding between the antigen and the antibody, cells stably expressing hKv1.3 channels were preincubated with the peptide-specific polyclonal antibody for two hours at 36°C and then superfused to wash off unbound antibodies, as undergone in immunoexperiments.

**Figure 3:**
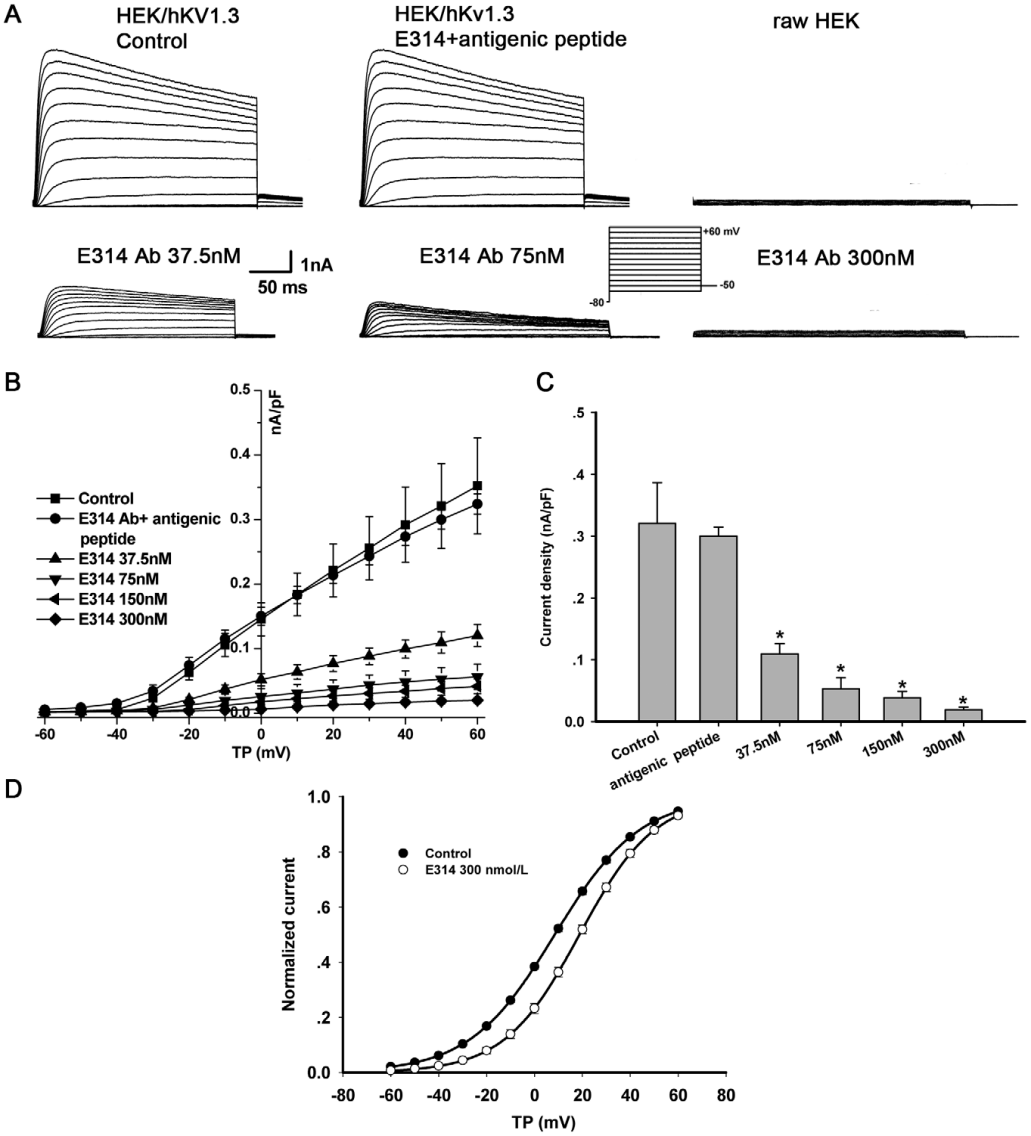
The E314 antibody inhibits human Kv1.3 currents stably expressed in HEK 293 cells. (A) I_Kv1.3_ traces were activated at 0.1 Hz upon 250 ms voltage steps to between −60 and +60 mV (10-mV increment) from −80 mV with tail current at −50 mV in HEK 293 cells stably expressing hKv1.3 currents. The current was absent in raw HEK 293 cells. (B) The E314 antibody inhibited hKv1.3 currents in a concentration-dependent manner at test potentials from −30 to +60 mV and the inhibition was abolished by the E314 antibody preincubated with an excess of the E314 peptide, as shown in I–V relationship. (C) At depolarizing pulse +50 mV, the E314 antibody ranging from 37.5 to 300 nM inhibited hKv1.3 current density respectively by 66% (*P*<0.001), 84% (*P*<0.001), 88% (*P*<0.001) and 94% (*P*<0.001). (D) Steady-state activation relationships of hKv1.3 channels were fitted to a Boltzmann distribution: y = 1/{1+exp[(Vm-V_0.5_)/S]}, where Vm is the membrane potential, V_0.5_ is the midpoint, and S is the slope. V_0.5_ for activation conductance of I_Kv1.3_ was 8.5±0.8 mV in control, and 18.7±1.0 mV in the 300 nM E314 antibody (n = 6, *P*<0.05). S was respectively 17.8±0.2 mV or 15.8±0.9 mV, for control and the 300 nM E314 antibody (*P*>0.05).

I_Kv1.3_ was elicited by the 300-ms voltage steps from −80 mV to between −60 and +60 mV (as shown in the inset) in HEK 293 cells stably expressing hKv1.3 channel and I_Kv1.3_ was absent in raw HEK 293 cells (Fig. 3A). The E314 antibody with a concentration of 300, 150,75, or 37.5 nM significantly decreased hKv1.3 current densities at test potentials from −30 to +60 mV and the inhibition was stronger at more positive potentials. The inhibition showed a concentration-dependence (Fig. 3B). At the depolarizing pulse +50 mV, the E314 antibody with concentrations ranging from 37.5 nM to 300 nM inhibited human Kv1.3 current densities respectively by 66%, 84%, 88% or 94% (0.10951±0.0165 nA/pF, 0.05285±0.01825 nA/pF, 0.03848±0.01049 nA/pF, 0.01914±0.0043 nA/pF, vs 0.32094±0.06573 nA/pF,*P*<0.001 vs control) (Fig. 3C).

To verify that the E314 antibody does bind to the external end of hKv1.3 pore region where the E314 peptide was generated and that the inhibiting effect is attributed to the binding of the E314 antibody to the hKv1.3 channel, we recorded hKv1.3 currents in the presence of the 300 nM E314 antibody that was preincubated with an excess of the E314 peptide. Supposed that supression was due to binding of the antibody to the peptide in external pore region, the inhibition should be prevented by preincubation with the peptide. As shown in Figure 3B and 3C, the inhibiting effect of the E314 antibody on I_Kv1.3_ was abolished after preincubation with the peptide, which indicated that the inhibition was due to specific binding of the E314 antibody to the E314 peptide around hKv1.3 pore region.

Voltage dependence of hKv1.3 channel activation (I/Imax) was determined by normalizing I_Kv1.3_ in the absence and presence of the 300 nM E314 antibody. Data were fitted to a Boltzmann distribution to obtain the half-activation voltage (V_0.5_) and the slope factor (S). The V_0.5_ of I_Kv1.3_ activation conductance was positively shifted by 10.2 mV (from 8.5±0.8 mV of control to 18.7±1.0 mV of the E314 antibody, n = 6, *P*<0.01) by the 300 nM E314 antibody, and the slope factor was slightly decreased (17.8±0.2 mV for control, 15.8±0.9 mV for the E314 antibody, *P*>0.05) (Fig. 3D). The E314 antibody affects the activation gating of hKv1.3 channels.

### The E314 antibody inhibits human Kv1.3 currents in Jurkat T cells

**Figure 4:**
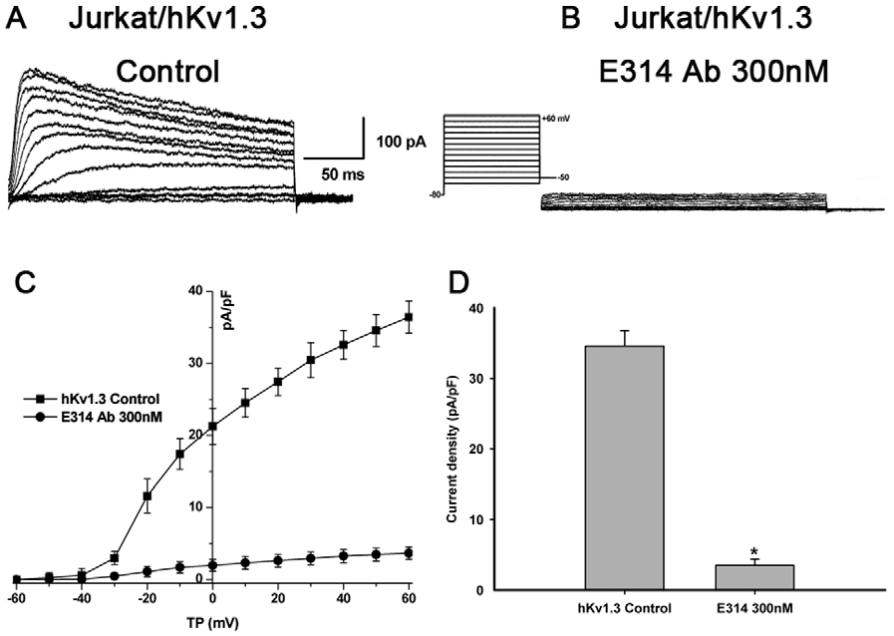
The E314 antibody inhibits I_Kv1.3_ in Jurkat T cells. I_Kv1.3_ expressed in Jurkat T cells was recorded with the voltage protocol as described previously in the absence (A) and presence (B) of the 300 nM E314 antibody. (C) I–V relationship of I_Kv1.3_ in the presence of the 300 nM E314 antibody at test potentials from −30 to +60 mV. (D) At depolarizing pulse +50 mV, the 300 nM E314 antibody inhibited hKv1.3 current densities by 90% (*P*<0.001).

To further study the ability of the E314 antibody inhibiting hKv1.3 currents, we also tested the effect of the anibody on human leukemia T cell line, Jurkat E6-1 cells. I_Kv1.3_ expressed in Jurkat T cells preincubated with the E314 antibody for two hours at 36°C was recorded with the voltage protocol as described previously in the absence and presence of the 300 nM E314 antibody (Fig. 4A and B). The 300 nM E314 antibody significantly decreased hKv1.3 current densities at test potentials from −30 to +60 mV and the inhibition was stronger at more positive potentials (Fig. 4C). At the depolarizing pulse +50 mV, the 300 nM E314 antibody inhibited hKv1.3 current densities by 90% (3.49552±0.89790 nA/pF vs 34.57908±2.21566 pA/pF,*P*<0.001) (Fig. 4D).

### The E314 antibody has no significant effect on I_Kv1.1_, I_Kv1.2_, I_Kv1.4_, I_Kv1.5_, I_KCa3.1_, I_HERG_, I_hKCNQ1/hKCNE1,_ I_CaL_ or I_Na_

**Figure 5:**
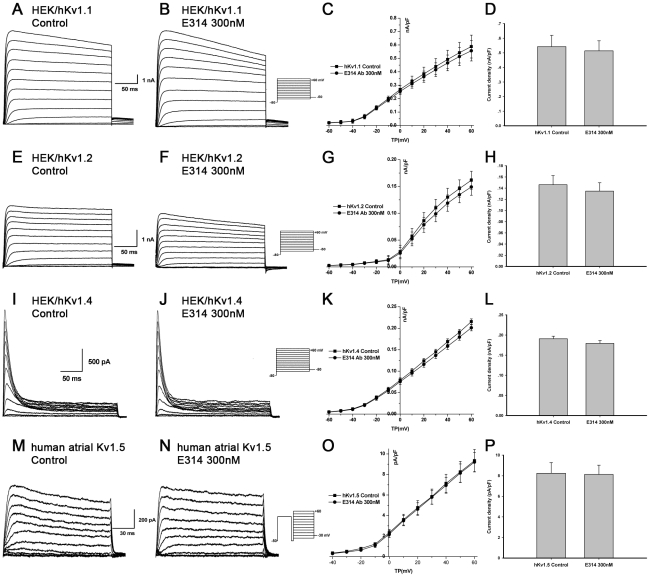
No significant effect of the E314 antibody on I_Kv1.1_, I_Kv1.2_, I_Kv1.4_ or I_Kv1.5_. I_Kv1.1_, I_Kv1.2_ and I_Kv1.4_ stably expressed in HEK 293 cells were recorded with the voltage protocol described as I_Kv1.3_ and I_Kv1.5_ was elicited at 1 Hz with a 100-ms prepulse to +40 mV to inactivate I_to1_, followed by 150-ms test pulses from −50 to between −40 and +60 mV after a 10-ms interval, then to −30 mV in human atrial myocytes. There was no pronounced alteration of I_Kv1.1_ (A, B and C), I_Kv1.2_ (E, F and G), I_Kv1.4_ (I, J and K) or I_Kv1.5_ (M, N and O) at all test potentials in the presence of the 300 nM E314 antibody. At depolarizing pulse +50 mV for I_Kv1.1_, I_Kv1.2_, I_Kv1.4_ or I_Kv1.5_, the E314 antibody caused no significant effect on I_Kv1.1_ (D), I_Kv1.2_ (H), I_Kv1.4_ (L) or I_Kv1.5_ (P) (*P*>0.05).

**Figure 6:**
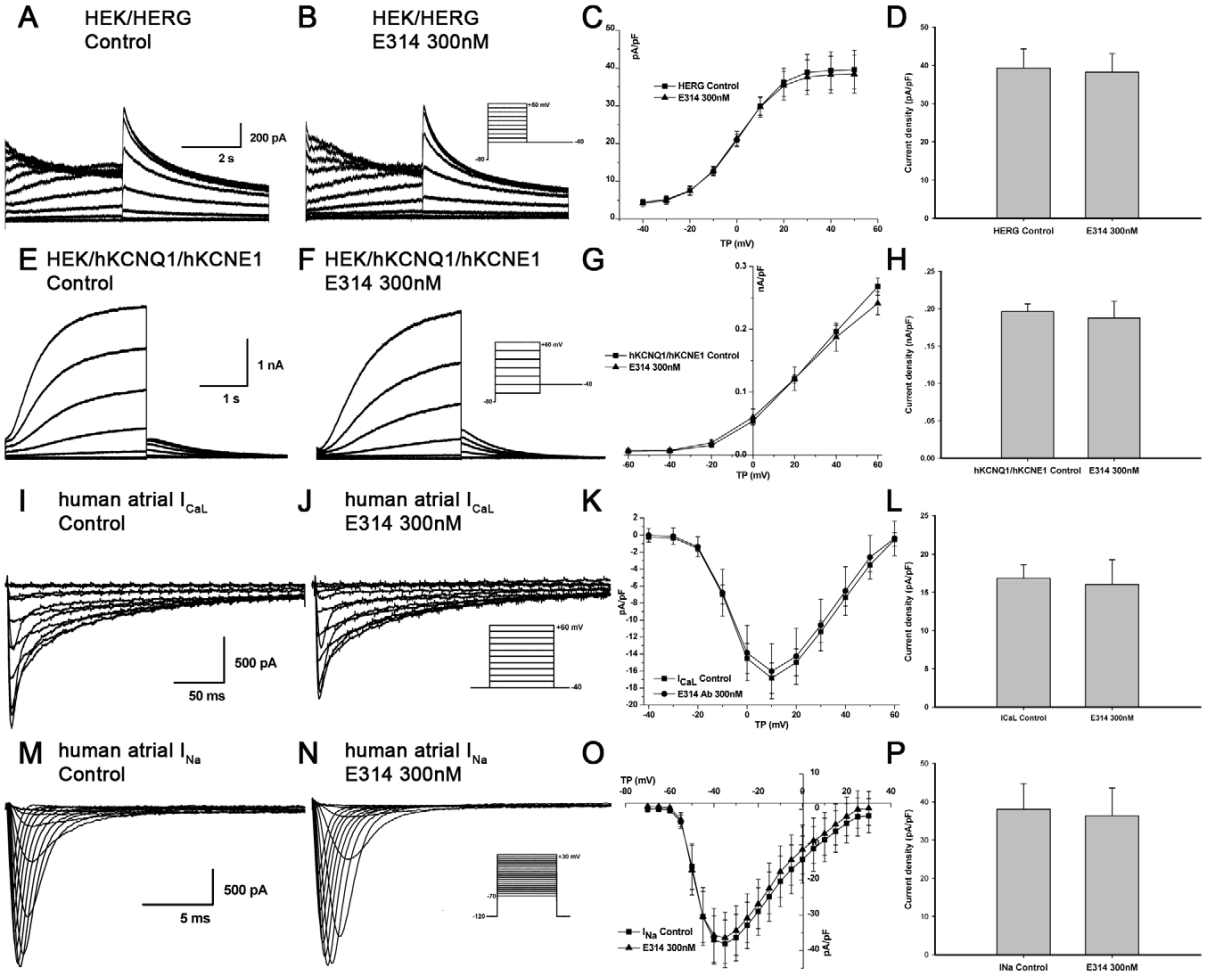
No significant effect of the E314 antibody on I_HERG_, I_hKCNQ1/hKCNE1_, I_Na_ or I_CaL_. I_HERG_, I_hKCNQ1/hKCNE1_ stably expressed in HEK 293 cells, I_Na_ and I_CaL_ in human atrial myocytes were recorded with the voltage protocol shown in the inset in the absence and presence of the 300 nM E314 antibody. There was no pronounced alteration of I_HERG_ (A, B and C), I_hKCNQ1/hKCNE1_ (E, F and G), I_CaL_ (I, J and K) or I_Na_ (M, N and O) at all test potentials in the presence of the 300 nM E314 antibody. At depolarizing pulse +40 mV for I_HERG.tail_, I_hKCNQ1/hKCNE1.step_, +10 mV for I_CaL_ or −35 mV for I_Na_, the E314 antibody caused no significant effect on I_HERG.tail_ (D), I_hKCNQ1/hKCNE1.step_ (H), I_CaL_ (L) or I_Na_ (P) (*P*>0.05).

**Figure 7:**
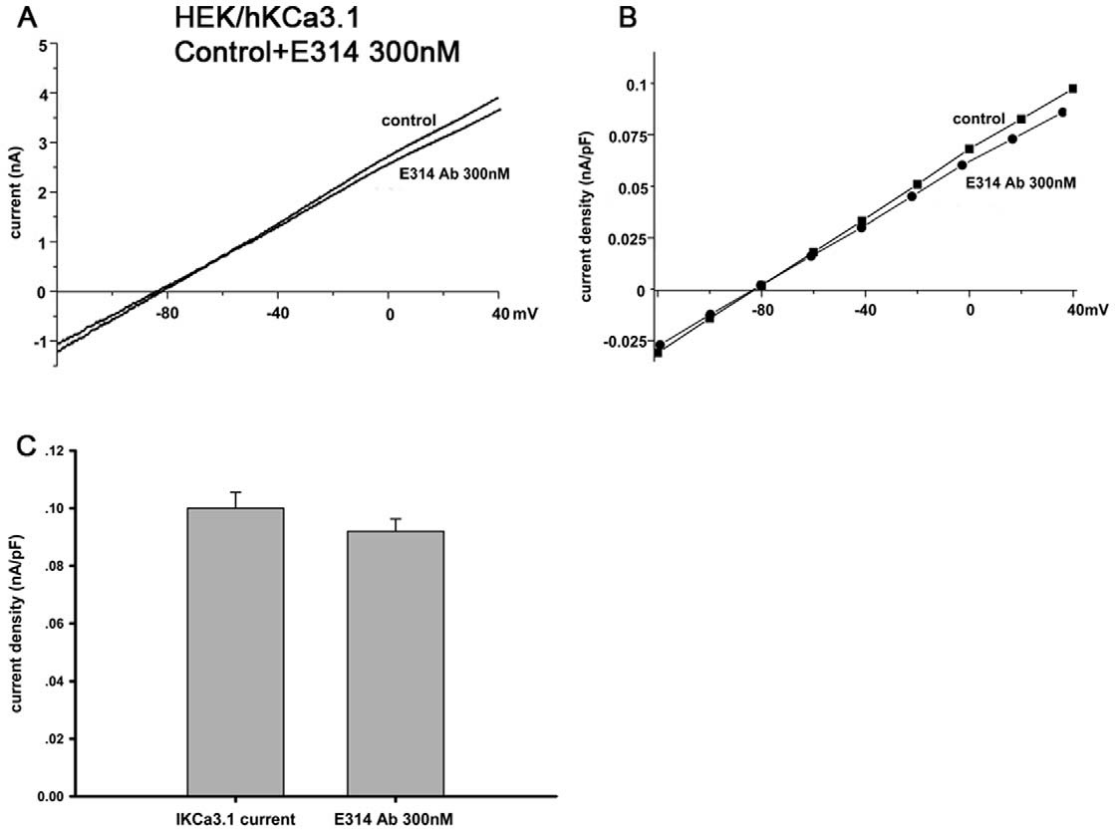
No significant effect of the E314 antibody on I_KCa3.1_. I_KCa3.1_ stably expressed in HEK 293 cells was elicited by 200-ms voltage ramps from −120 to 40 mV applied every 10 s in the absence and presence of the 300 nM E314 antibody. There was no pronounced alteration of I_KCa3.1_ (A and B) at all test potentials in the presence of the 300 nM E314 antibody. At depolarizing pulse +40 mV, the E314 antibody caused no significant effect on I_KCa3.1_ (D) (*P*>0.05).

To further verify the specificity of the E314 antibody, we examined the effect of E314 antibody on other closely related K_v_1-family channels, functional cardiac ion channels and KCa3.1 channel which is another important potassium channel expressed in T lymphocytes. Human atrial myocytes were used to observe the effect of E314 on I_Kv1.5_, I_CaL_ or I_Na_ and the effect of E314 on I_Kv1.1_, I_Kv1.2_, I_Kv1.4_, I_KCa3.1_, I_HERG_ or I_hKCNQ1/hKCNE1_ was studied in HEK 293 cells stably expressing hKv1.1 channel, hKv1.2 channel, hKv1.4 channel, hKCa3.1 channel, HERG channel or hKCNQ1/hKCNE1 channels. Voltage-dependent I_Kv1.1_, I_Kv1.2_, I_Kv1.4_, I_Kv1.5_, I_KCa3.1_, I_HERG_, I_hKCNQ1/hKCNE1_, I_CaL_ or I_Na_ were elicited respectively by the voltage protocol shown in the inset of Figure 5, 6 and 7. In contrast to its effect on hKv1.3 currents, exposure of the 300 nM E314 antibody yielded limited effects on I_Kv1.1_, I_Kv1.2_, I_Kv1.4_, I_Kv1.5_, I_KCa3.1_, I_HERG_, I_hKCNQ1/hKCNE1_, I_CaL_ or I_Na_ at all test potentials. At the depolarizing pulse +50 mV for I_Kv1.1_, I_Kv1.2_, I_Kv1.4_ and I_Kv1.5_, +40 mV for I_KCa3.1_, I_HERG.tail_ and I_hKCNQ1/hKCNE1.step_, −35 mV for maximal I_Na_, +10 mV for maximal I_CaL_, the 300 nM E314 antibody caused no statistically significant effect on peak value of each current including I_Kv1.1_ (0.51±0.07 nA/pF vs 0.54±0.08 nA/pF, *P*>0.05), I_Kv1.2_ (0.13±0.02 nA/pF vs 0.15±0.02 nA/pF, *P*>0.05), I_Kv1.4_ (0.18±0.01 nA/pF vs 0.19±0.01 nA/pF, *P*>0.05), I_Kv1.5_ (8.13±0.90 pA/pF vs 8.25±1.03 pA/pF, *P*>0.05), I_KCa3.1_ (0.09 nA/pF vs 0.10 nA/pF, *P*>0.05), I_HERG.tail_ (38.38±5.02 pA/pF vs 39.58±5.13 pA/pF, *P*>0.05), I_hKCNQ1/hKCNE1.step_ (0.24±0.02 nA/pF vs 0.27±0.01 nA/pF, *P*>0.05), I_CaL_ (16.03±3.23 pA/pF vs 16.84±1.8 pA/pF, *P*>0.05) and I_Na_ (37.33±6.67 pA/pF vs 38.55±6.73 pA/pF, *P*>0.05). (Fig. 5, 6 and 7).

## Discussion

Autoimmune diseases or autoimmune-associated diseases afflict millions of people in the world. Overreaction of immune activities plays a pivotal role in the pathogenesis of these diseases [11]. Thus immunosuppression therapy is of broad use in the management of autoimmune diseases [20], [38]. However pleiotropic actions of immunosuppressants clinically available such as methylprednisolone limit therapeutic values [39], [40]. For patients, more specific immunosuppression would help to ameliorate disease with less adverse or side effects [24], [41]. In recent years, selective blockade of Kv1.3 in T_EM_ cells has exhibited the potential of specific inhibition of T lymphocyte subsets and leaves protective immunity unharmed, which encourages increasing efforts to explore selective or specific Kv1.3 blockers [36], [41]–[43]. In this study, we presented a novel, potent and specific Kv1.3 blocker.

Unlike the heteromultimeric Kv1.3 channel expressed in neurons, Kv1.3-containing homotetramers in lymphocytes comprise 4 identical subunits [44], [45]. We expressed the identical subunits in a stable HEK 293 cell lines, which represents Kv1.3 channels expressed in lymphocytes. The antibody designed targeting one subunit would be possible to exert its effect on the Kv1.3 channel. By computing the antigenic index and hydrophilicity of hKv1.3 constituent amino acid aligment, we selected an antigenic peptide that is located at the external end of the pore region which has been successfully used to generate polyclonal antibodies against several ion channels [46], [47]. The peptide location was indicated by immunostaining of the E314 antibody binding. The pore region is the way mediating K^+^ efflux and determines ion permeability [48], [49]. The E314 antibody blocks the pore region from the external end with a large interacting surface, which utilizes a “cork in the bottle” strategy exemplified by peptide toxins, covering and plugging the external end [24], [42]. The external block by the E314 antibody yields the K^+^ impermeability. In addition, the external block is stable, in contrast with the internal block by small molecules which is greatly affected by gating-associated conformational changes and state-dependent [37], [50]. The external block gives the E314 antibody more potency. In this study, the E314 antibody in nanomolar concentrations showed a strong inhibition, comparable to some selective kv1.3 blockers [38], [51], [52].

In clinics, a variety of drugs including immunosuppressants can increase the risk of drug-acquired arrhythmias due to the impact on functional cardiac ion channels [53]–[56]. It is documented that drug binding sites among Kv1.3, Kv1.5, HERG and hKCNQ1/hKCNE1 channels are conservative [21], [36], [43], [57]. The conservation hinders some Kv1.3-blocking candidates from being developed into good drugs. Thus, to prevent the occurrence of drug-acquired arrhythmias, the ICH E14 and S7B guidance issued by FDA calls for the assessment of the potential of any drug to delay cardiac repolarisation [58]–[60]. Besides, loss function of closely related Kv1 channels, such as Kv1.1 or Kv1.2 is able to result in CNS disorders [61], [62]. By blast, we found that there is a maximal homology between the hKv1.3-E314 peptide and the corresponding peptides of hKv1.1, hKv1.2, hKv1.4 or hKv1.5. In addition KCa3.1 channels in T lymphocytes might be of importance for immune-mediated side-effects [63]. Recently the tandem of pore domains in a weak inwardly rectifying potassium channel-related acid-sensitive potassium channels (TASK1–3) have been found to be expressed in the nervous system, T lymphocytes and heart and play important role in cardiac repolarization, autoimmune inflammation, cancer development and CNS disorders [64]–[66]. Therefore, this study was necessary to be focused on the effect of the E314 antibody as a potential candidate for specific Kv1.3 blockers on functional cardiac ion channels, closely related Kv1 channels, KCa3.1 channels and TASK1–3 channels.

Immunoexperiment results indicate that the E314 antibody can specifically recognize hKv1.3 protein, whereas it is not able to recognize or cross-react to hKv1.1, hKv1.2, hKv1.4, hKv1.5, hKCa3.1, HERG, hKCNQ1/hKCNE1 or human Na_v_1.5, Ca_v_1.2 proteins. By the patch clamp technique, we demonstrated that the E314 antibody with a high concentration exhibits no significant effect on these closely related K_v_1 channels, KCa3.1 channels or functional cardiac ion channels. By blast analysis, we found that there is no homology between Kv1.3 and TASK1–3 channels. All the results indicate that the E314 antibody is able to function as a novel specific hKv1.3 blocker without worries about its potential proarrhythmias, immune-mediated side-effects or CNS disorders, which is required as a safe clinic-promising channel blocker.

Based on the E314 antibody generation regimen, one monoclonal antibody with more affinity and specificity or a vaccine targeting hKv1.3 E314 peptide, which can serve as a novel hKv1.3 blocker to inhibit autoreactive T lymphocyte activities, would probably be developed as a novel drug for the treatment of autoimmune diseases or autoimmune-associated diseases. The attractiveness arises from the excellent track record of several monoantibodies and a vaccine, such as infliximab [67], adalimumab [68], natalizumab [69], Anti-IL-17A vaccine [70], which is typical of targeting biological therapy, gradually superior to traditional immunosuppressants.

## Materials and Methods

### Ethics statement

In this study, the procedure of obtaining human atrial and ventricle specimens from patients receiving cardiac surgery conforms to the principles outlined in the Declaration of Helsinki. The study was approved by the Ethics Committee of Tongji Medical College of Huazhong University of Science and Technology and patients provided written informed consent.

In this study, the animal use and care protocol was approved by the Ethical Committee on Animal Experimentation of Tongji Medical College, Huazhong University of Science and Technology (Approval ID: 00009678). All surgery was performed under sodium pentobarbital anesthesia, and every effort was made to minimize suffering.

### Antibody generation

One extracellular peptide located at E3 loop between hKv1.3 spanning S5 and S6 was selected as an antigenic determinant according to its constituent amino acid antigenic index calculated by the software DNAStar and the corresponding amino acid alignment was synthesized by PSSM8 Peptide Synthesizer (Shimadzu, Japan). Adult male New Zealand white rabbits from the Center of Experimental Animals (Tongji Medical College, Huazhong University of Science and Technology, China) were fortnightly immunized by subcutaneous injection of a mixture of the synthetic peptide conjugated with bovine serum albumin (BSA) and complete or incomplete Freund's adjuvant. Rabbits receiving sham immunization with a mixture of physiological saline conjugated with complete or incomplete Freund's adjuvant were as a control. Serum was collected before each immunization and the rabbits were sacrificed on 63 d of the experiment. Harvested serum were screened by enzymelinked immunosorbent assay (ELISA) and purified on a protein A column (UNOsphere SUPrA Affinity Cartridge, Bio-Rad, US). The E314 antibody was additionally purified on an peptide-affinity column (GL Biochem Ltd, Shanghai, China).

### Cell culture and establishment of cell lines

HEK 293 cells were purchased from the American Type Culture Collection (ATCC Manassas, VA, USA) and grown in Dulbecco's modified Eagle medium (DMEM, Invitrogen, Carlsbad, CA) supplemented with 10% fetal bovine serum (Invitrogen, Carlsbad, CA) in 5% CO_2_ and 95% air at 37°C.

Jurkat-T cells (clone E6-1) were purchased from the China Center for Type Culture Collection (CCTCC Wuhan, China) and grown in RPMI 1640 media (Invitrogen, Carlsbad, CA) supplemented with 10% fetal bovine serum (Invitrogen), 1 mM sodium pyruvate (Invitrogen) and 1% penicillin/streptomycin (Invitrogen) in 5% CO_2_ and 95% air at 37°C.

The vector of hKv1.3/pCI-neo generously provided by Dr. Garcia Maria (Merck & Co. Inc, West Point, PA) was transfected into HEK 293 cells using FuGENE® HD (Roche, Germany) following the manufacturer's instructions. The HEK 293 cell line stably expressing hKv1.3 channels were selected in 800 µg/ml G418 (Invitrogen, Carlsbad, CA) and was maintained in DMEM containing 400 µg/ml G418.

HEK 293 cell lines stably expressing hKv1.1, hKv1.2, hKv1.4, HERG or hKCNQ1/hKCNE1, KCa3.1 channels were established in a similar way. The vector of HERG/pcDNA3 generously provided by Dr. G. Robertson (University of Wisconsin, Madison, WI, USA) was transfected into HEK 293 cells using Attractene (QIAGEN, US). The vector of hKCNQ1/pCEP4 was a generous gift from Dr. GR Li (University of Hong Kong, Pokfulam, Hong Kong, China) and human KCNE1, Kv1.1, Kv1.2 and Kv1.4, KCa3.1 cDNAs, synthesized by the corporation of FulenGene (China) were subcloned into pcDNA3 vector (Invitrogen, CA). The vectors of hKCNQ1/pCEP4 and hKCNE1/pcDNA3 were co-transfected into HEK 293 cells using Attractene (QIAGEN, US). The vectors of hKv1.1/pcDNA3, hKv1.2/pcDNA3, hKv1.4/pcDNA3 and hKCa3.1/pcDNA3 were transfected into HEK 293 cells using Attractene (QIAGEN, US) seperately. After selection in 800 µg/ml G418 or 200 µg/ml hygromycin (Roche, Germany), HEK 293 cell lines stably expressing hKv1.1, hKv1.2, hKv1.4, hKCa3.1, HERG or hKCNQ1/hKCNE1 channels were maintained in DMEM containing 400 µg/ml G418 or 100 µg/ml hygromycin.

### Human atrial myocyte isolation

Atrial myocytes were isolated from specimens of human right atrial appendage obtained from patients receiving cardiac surgery aging from 25 to 70 years old (male 6, female 5). After excision, the samples were quickly immersed in oxygenated, Ca^2+^-free cardioplegic solution for transport to the laboratory. One single atrial myocyte was enzymatically dissociated under the sterile circumstance. Briefly, the atrial tissue was minced and the pieces were digested first in a mixture of 150–200 U/ml collagenase (CLS II, Worthington Biochemical, Freehold, NJ, U.S.A.), 1.2 U/ml protease (type XXIV, Sigma Chemical, St Louis, MO, U.S.A.), and 1 mg/ml bovine serum albumin (Sigma-Aldrich, St. Louis, MO) for 50 minutes and then in a Ca^2+^-free Tyrode solution with the same composition, but without protease for about 30 minutes after being washed three times in a Ca^2+^-free Tyrode solution at 36°C and gently agitated by continuous bubbling with 100% O_2_. The isolated atrial myocytes were kept in a High-K^+^ storage solution at room temperature for at least 1 hour until use.

### Immunofluorescence

HEK 293 cells or human atrial myocytes were stained by immunofluorescence for the detection of the binding of the E314 antibody to plasma membranes. After adherent cells were fixed and blocked with 10% BSA and 1% normal donkey serum, these cells were incubated with the E314 antibody diluted at 1∶200 at 4°C overnight and then with FITC-labelled donkey anti-rabbit secondary antibodies (Chemicon International) at room temperature for 2 h away from light. The cells were observed in a Olympus FluoView™ FV1000 (Olympus, Japan) laser-scanning confocal microscope. In control experiments, the primary antibody were preincubated with an excess of E314 antigenic peptide as a negative control.

### Western blotting

To confirm the E314 antibody specific recognition, Western-blotting analysis was performed. Human atrial or ventricular specimens and HEK 293 cells were lysed and proteins were extracted. Proteins were analyzed by electrophoresis in 7.5% (w/v) polyacrylamide gel containing 0.1% (w/v) sodium dodecylsulfate (SDS) followed by blotting to a nitrocellulose membrane. After blocking, the membrane was incubated with the E314 antibody diluted at 1∶1000 overnight at 4°C and then incubated with horseradish peroxidase-conjugated anti-rabbit IgG antibody 2 h at room temperature. The blot was visualized by a chemiluminescence's method (ECL Western blotting detection system). In control experiments, the primary antibody were preincubated with an excess of E314 antigenic peptide as a negative control.

### Electrophysiological recording solution and drugs

Ca^2+^-free cardioplegic solution for specimen transport contained (in mM): KH_2_PO_4_ 50, MgSO_4_ 8, adenosine 5, HEPES 10, glucose 140, mannitol 100, taurine 10, and pH was adjusted to 7.3 with KOH. High-K^+^ storage solution contained (in mM): KCl 10, K-glutamate 120, KH_2_PO_4_ 10, MgSO_4_ 1.8, taurine 10, HEPES 10, EGTA 0.5, glucose 20, mannitol 10, pH adjusted to 7.3 with KOH. The standard Tyrode solution contained (in mM): NaCl 140, KCl 5.4, MgCl_2_ 1, CaCl_2_ 1, NaH_2_PO_4_ 0.33, HEPES 10, glucose 10, pH was adjusted to 7.4 with NaOH. The pipette solution contained (in mM): KCl 20, K-aspartate 110, MgCl_2_ 1, HEPES 10, EGTA 5, GTP 0.1, Na_2_-phosphocreatine 5, and Mg-ATP 5, pH was adjusted to 7.2 with KOH. For perforated-patch recordings, β-escin (Sigma-Aldrich, St. Louis, MO) was added to the pipette solution (0.042 µg/ml). For I_Kur_ recording, BaCl_2_ (200 µM) and CdCl_2_ (200 µM) were added to the superfusion to block I_K1_ and I_Ca.L_. For I_Ca.L_ recording, K^+^ in pipette and Tyrode solution was replaced by CsCl. I_Na_ was recorded under K^+^-free conditions with symmetrical Na^+^ (5 mM) in pipette and superfusion solutions. For I_KCa3.1_ the pipette solution contained (in mM): K-aspartate 145, MgCl_2_ 2, HEPES 10, EGTA 10, CaCl_2_ 8.5, pH was adjusted to 7.2 with KOH and the external solution contained (in mM): Na-aspartate 160, KCl 4.5, CaCl_2_ 2, MgCl_2_ 1, HEPES 5, pH was adjusted to 7.4 with NaOH.

### Patch-clamp recording

HEK 293 cell lines, Jurkat T cells or human atrial myocytes were incubated at least 2 h with the E314 antibody at 36°C for total binding of the E314 antibody. Borosilicate glass electrodes (1.2-mm OD) were constructed using a Brown-Flamming puller (model P-97, Sutter Instrument Co, Novato, CA, USA) and had tip resistances of 2–3 MΩ when filled with pipette solution for I_Kv1.3_, I_Kv1.1_, I_Kv1.2_, I_Kv1.4_, I_Kv1.5_, I_KCa3.1_, I_HERG_, I_hKCNQ1/hKCNE1_ or I_CaL_ recording and 0.5–1 MΩ for I_Na_ recording. Membrane currents were recorded using an Axopatch200B amplifier and Clampex software (Molecular Devices, USA). For I_Kv1.3,_ I_Kv1.1_, I_Kv1.2_, I_Kv1.4_, I_Kv1.5_, I_KCa3.1_, I_HERG_ or I_Na_ recording, whole -cell patch clamp was used and perforated patch configuration was performed for I_hKCNQ1/hKCNE1_ or I_CaL_ recording with additional 0.042 µg/ml Escin (Sigma-Aldrich) in the pipette solution. Electrical signals were low-pass filtered at 5 kHz and stored on the hard disk of computer for off-line analysis using clamfit or sigmaplot.

### Statistical Analysis

Data were expressed as mean±SEM. Statistical analysis was performed using SPSS 12.0 software. Differences between groups were analyzed by *t* test or ANOVA. *P* value less than 0.05 was considered statistically significant.

## Supporting Information

Figure S1 **Time course of the blockage of I_Kv1.3_ by the E314 antibody.** Time course of I_Kv1.3_ was recorded with 250 ms test pulses from −80 to 50 mV. The Kv1.3 currents amplitude reduced about 55% after addition of the 300 nM E314 antibody in 10–15 minutes and the inhibition was not reversible by washout.
(TIF)
