# Supplementary figures and images for: The Antibody Targeting the E314 Peptide of Human Kv1.3 Pore Region Serves as a Novel, Potent and Specific Channel Blocker
Source: PLoS One. 2012 Apr 27;7(4):e36379. doi: 10.1371/journal.pone.0036379 (PMC3338681; doi:10.1371/journal.pone.0036379)

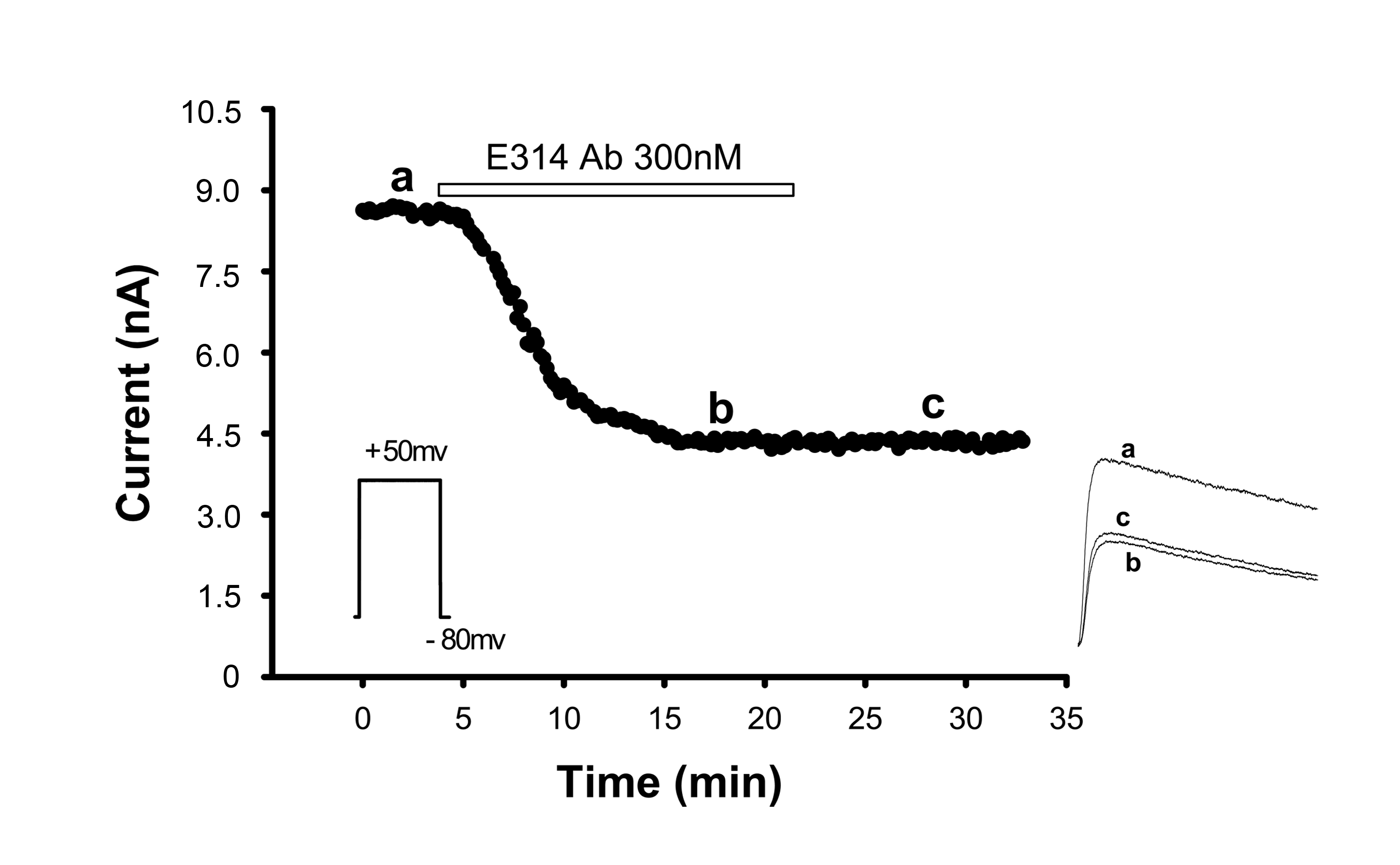

Supplement: Figure S1 — Time course of the blockage of IKv1.3 by the E314 antibody. Time course of IKv1.3 was recorded with 250 ms test pulses from −80 to 50 mV. The Kv1.3 currents amplitude reduced about 55% after addition of the 300 nM E314 antibody in 10–15 minutes and the inhibition was not reversible by washout. (TIF) [file pone.0036379.s001.tif]
